# Affordable innovation: future directions in pharmaceutical policy

**DOI:** 10.1186/2052-3211-8-S1-K1

**Published:** 2015-10-05

**Authors:** Suzanne R Hill

**Affiliations:** 1 Department of Essential Medicines and Health Products, World Health Organization (WHO), 1211 Geneva 27, Switzerland

## 

In 2009, Jayadev and Stiglitz [1] proposed ‘two ideas’ to increase innovation and reduce pharmaceutical costs and prices. These were the use of value-based pricing and promoting public funding of clinical trials. Since then, NICE has tried and not yet succeeded to introduce value-based pricing, Persson and Jonsson [2] suggest that international reference pricing should cease, Gilead has made billions on the basis of exorbitant pricing of new products for hepatitis C, and prices for new products for cancer are causing concern even in the US [3]. At the same time, there has been considerable work on how to promote research and development to meet health needs in developing countries [4], the reported cost of drug development seems to continue to increase [5], the structure of the multinational industry has changed significantly [6], there are major area of market failure such as antibiotics, and investment in public-private partnerships to promote innovation continues to grow.

Given this complex landscape, this abstract briefly reviews the current ideas and strategies to promote innovation in pharmaceutical development. It provides a review of some of the issues about pricing and affordability of new medicines, and some options for ensuring that innovation is affordable. These options are likely to require some significant changes in our current approaches to negotiating pharmaceutical prices with suppliers, increased global and regional collaboration as well as increased transparency about clinical effects that are relevant to patients, costs of research and development and explicit consideration of willingness to pay by different countries.

The problem of declining innovation in the pharmaceutical industry has been well described [6]. Trouiller et al. summarised the problem in 2002 [7], showing how few molecules had been approved by the FDA that were of relevance to neglected tropical diseases. The Priority Medicines for Europe and the World Report in 2004 [8] was one response, to identify priority products for potential development by European pharma. The regulatory framework for orphan drugs in both the USA and Europe was another mechanism [9] and similarly, the push for paediatric medicines tried to use incentives (patent protection) as well as sanctions to encourage needed product development.

In 2012, the report [4] by the Consultative Expert Working Group on Research and Development identified a group of proposals that they considered most likely to promote innovation in research for products relevant to low and middle income countries. These were: a ‘Global Framework’ on research and development, open approaches to research and development and innovation, pooled funds, direct grants to companies, milestone prizes and end prizes and patent pools. They made recommendations on how much they thought countries should contribute to financing research and development activities, and also suggested that the WHO should take a coordinating role, including a global health research and development observatory.

Another example of promoting innovation may be the increasing number of public-private partnerships. There are the well-established entities, such as MMV and DNDi who have a portfolio of products for malaria and neglected tropical disease respectively. New products from both are on the market. Another venture, the Innovative Medicines Initiative is the biggest public-private partnership to date, and is also hoping to promote the development of innovative medicines.

## But what about the price of the new products?

Prices of new medicines are a global problem. In the USA, prices of new cancer medicines have risen in real terms by 10% per year since 2005 [10], such that the entry price of recent new molecules – for example, ipilimumab for melanoma – is now estimated to be USD120,000/patient/course of treatment – approximately twice the average annual income. Some perverse incentives in the US legislation maybe contributing to the high prices, but mostly they appear to be due to ‘what the market will pay’. So the medicines for hepatitis C, sofosbuvir and the combination with ledipasvir, marketed by Gilead, have public prices of USD1,000 per pill in high income countries. We have estimated that, at this price, the cost of treating even a small proportion of the total Hep C infected patient population is unaffordable for most high income countries. Even medicines for orphan disease, where arguably higher prices might be justified on the basis of small market volumes are setting new price records, despite public funding of a significant proportion of the development costs in some cases for example, ivacaftor for cystic fibrosis [11].

In most high income countries, a number of policies are used to manage prices of medicines and expenditure. The choice of policy has to be set in the context of the balance between health and industrial imperatives, but usually includes some, or all, of: price-setting techniques (reference pricing, profit ceilings, cost-plus pricing and value-based pricing), control of supply chain prices and mark-ups from ex-manufacturer to dispensing, managing purchasing (through lists, tenders, price volume agreements and pooling procurement) and price signals, through co-payments, or premiums to promote generic completion and prescribing.

In low and middle income countries, although medicine prices are often reported to be high, there is less control of the supply chain and use of price setting techniques. As a result, especially in countries without comprehensive coverage or insurance, out of pocket payments can be catastrophic for individuals. While direct evidence of the effect of pricing policies in LMIC is limited [12], it would seem reasonable to assume that controlling prices and mark-ups would have the same effect on price as it does in high income countries. How to take control of a market is a much more difficult question.

Newer approaches to managing prices are also developing. A number of high income countries are using policies such as ‘risk sharing’, ‘managed entry’, ‘pay for performance’ and ‘coverage with evidence development’ [13,14]. The impact on access and prices these types of schemes will have is not clear, but there are certainly questions to consider: for example, if measurement of patient outcomes is required for payment, does that require a separate registry for each disease? And if so, how are the data managed and analysed? Who decides what will be measured? Who protects patient privacy and how?

Similarly, the use of confidential rebates and discounts appears to be increasing [2] such that the public prices listed are almost meaningless. If prices are negotiated behind closed doors, what principles are used to ensure an appropriate or fair price and how should it be done? Cost-effectiveness thresholds are one approach but can have a distorting effect if budget impact and affordability are not explicitly considered.

But the public anger over what is perceived as corporate greed [15] is likely to push for at least one change, which is much more transparency about the basis for pricing. GSK has provided one recent example of transparent cost-plus pricing, for their new malaria vaccine. Luzzatto et al. [9] suggest the same principle should apply to prices for medicines for orphan diseases. Differential pricing has been tried by Gilead for its products for hepatitis C, but without consideration of budget impact, which has been proposed as an alternative [16] or countries' willingness to pay. So to be able to afford innovation, we need to change our approach and bring all of these strategies together: we need to consider international price negotiation, with fair profit margins and transparent understanding of all production costs, as well as quality use of medicines. Stiglitz may be right about value-based pricing as a solution – but the challenge is how to get there.
